# Electronic Structure and Core Spectroscopy of Scandium Fluoride Polymorphs

**DOI:** 10.1021/acs.inorgchem.2c04357

**Published:** 2023-03-01

**Authors:** Fabiana Machado Ferreira de Araujo, Daniel Duarte-Ruiz, Holger-Dietrich Saßnick, Marie C. Gentzmann, Thomas Huthwelker, Caterina Cocchi

**Affiliations:** § Institute of Physics, Carl-von-Ossietzky Universität Oldenburg, 26129 Oldenburg, Germany; ⊥ Bundesanstalt für Materialforschung und-prüfung, Unter den Eichen 87, 12205 Berlin, Germany; ∥ Swiss Light Source (SLS), Paul Scherrer Institut (PSI), 5232 Villigen, Switzerland; ¶ Physics Department and IRIS Adlershof, Humboldt-Universität zu Berlin, 12489 Berlin, Germany

## Abstract

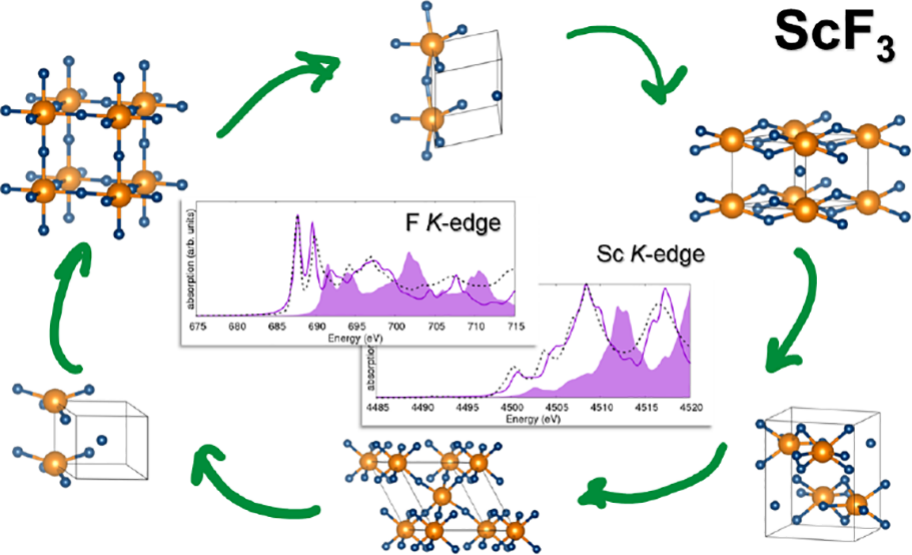

Microscopic knowledge of the structural, energetic, and electronic properties of scandium fluoride is still incomplete despite the relevance of this material as an intermediate for the manufacturing of Al–Sc alloys. In a work based on first-principles calculations and X-ray spectroscopy, we assess the stability and electronic structure of six computationally predicted ScF_3_ polymorphs, two of which correspond to experimentally resolved single-crystal phases. In the theoretical analysis based on density functional theory (DFT), we identify similarities among the polymorphs based on their formation energies, charge-density distribution, and electronic properties (band gaps and density of states). We find striking analogies between the results obtained for the low- and high-temperature phases of the material, indirectly confirming that the transition occurring between them mainly consists of a rigid rotation of the lattice. With this knowledge, we examine the X-ray absorption spectra from the Sc and F K-edge contrasting first-principles results obtained from the solution of the Bethe–Salpeter equation on top of all-electron DFT with high-energy-resolution fluorescence detection measurements. Analysis of the computational results sheds light on the electronic origin of the absorption maxima and provides information on the prominent excitonic effects that characterize all spectra. A comparison with measurements confirms that the sample is mainly composed of the high- and low-temperature polymorphs of ScF_3_. However, some fine details in the experimental results suggest that the probed powder sample may contain defects and/or residual traces of metastable polymorphs.

## Abstract 2

The stability and electronic structure of six ScF_3_ crystalline polymorphs are calculated from first principles using density functional theory. With the aid of X-ray spectroscopy measurements on a powder sample, the fingerprints of the material from the Sc and F K-edges are identified and contrasted against the solution of the Bethe−Salpeter equation on the computationally predicted structures, which enables the assessment of excitonic effects in the spectra.

## Introduction

The highly valuable transition-metal scandium (Sc) is suitable for various high-tech applications in solid oxide fuel cells, Al–Sc alloys, and the laser industry.^1−3^ Due to its dispersive nature, it is currently recovered as a byproduct of titanium dioxide, zirconium dioxide, uranium, and nickel production.^4^ In addition, Sc recovery from bauxite residues is under investigation or implemented on a pilot-plant scale in several locations.^5−7^ The ionic crystal scandium fluoride (ScF_3_) is an important intermediate compound for the manufacturing of Al–Sc alloys, where it is preferred over scandium oxide (Sc_2_O_3_).^8^ The use of Sc in Al alloys strongly enhances the properties of the material because it positively affects grain refinement, precipitation hardening, and superplasticity and increases recrystallization and corrosion resistance. ScF_3_ can be produced by solvent extraction from a Sc-containing solution followed by stripping and precipitation in the form of a hydroxide or an oxalate salt. The precipitate is first calcined to obtain Sc_2_O_3_ and subsequently fluorinated with hydrofluoric acid (HF) to finally obtain ScF_3_. Alternative methods to directly obtain ScF_3_ without using HF have recently been developed.^9^

From a fundamental viewpoint, ScF_3_ has received considerable interest in the past few years because of its negative thermal expansion, namely, its remarkable ability to shrink when heated.^10−17^ This property is closely related to the electronic structure of this material and the crystallographic arrangement of the two atomic species therein: Upon heating, F atoms oscillate around their bonds, with Sc leading to an overall contraction of the crystalline volume.^11^ The structural flexibility of this material is prone to polymorphism. At low-temperature and low-pressure conditions, ScF_3_ crystallizes in a cubic lattice of space group *Pm*3̅*m* with hexacoordinated Sc atoms, forming corner-sharing octahedra.^10^ At increasing temperature and/or pressure, the material undergoes a phase transition and assumes a trigonal configuration (space group *R*3̅*c*) in which the octahedra are rotated around their axes.^10,18^

Beyond the above-cited studies on the peculiar structural and thermal properties of ScF_3_, knowledge of the fundamental properties of this material is still incomplete. Questions regarding the stability of the crystal and its polymorphism, as well as the charge distribution within the lattice, demand answers for a deeper understanding of the microscopic characteristics of ScF_3_. This information will provide further insight not only into the intrinsic properties of this material but, more generally, on the electronic structure of Sc-based compounds. This fundamental knowledge is, furthermore, crucial for the development of applications that take advantage of the special properties of ScF_3_ such as its negative thermal expansion.

In this paper, we present a joint theoretical and experimental study to gain insight into the structure–property relationships in ScF_3_. In the framework of density functional theory (DFT), we investigate six ScF_3_ polymorphs, including the experimentally resolved low- and high-temperature phases^10,18^ as well as four computationally predicted structures,^19^ and perform a systematic analysis evaluating bond lengths, formation energies, partial charges, and electronic properties. These results are the baseline to interpret X-ray absorption spectra measured from the Sc and F K-edges on a single powder sample of ScF_3_. Analysis of the spectra computed from first principles by solving the Bethe–Salpeter equation (BSE) enables one to relate spectral fingerprints with the electronic structure of the material polymorphs, and it provides valuable information regarding the excitonic effects. A systematic comparison between the computed spectra and the measurements suggests that the powder sample probed in the experiments is predominantly made of the known stable phases of the material. Spectroscopic analysis of the polymorphs that are to date only computationally predicted enriches the established knowledge on ScF_3_ and provides the community with additional insight into the electronic structure of this compound in relation to the stability, the charge distribution, and the electronic properties of its different crystal structures.

## Methodology

### Theoretical Background and Computational Details

The six structures considered in the computational analysis are taken from *Materials Project*(19) (Table S1), and they are not further relaxed. The ground-state properties of the considered ScF_3_ polymorphs are calculated from DFT using the plane-wave, pseudopotential code *Quantum ESPRESSO*.^20^ In all cases, the Brillouin zone is sampled by a homogeneous 8 × 8 × 8 **k** grid. To ensure converged results, cutoff values of 150 and 1200 Ry for the plane waves and the density are chosen, respectively. The projector-augmented-wave method is applied to account for the core electrons using pseudopotentials from the pslibrary.^21^ The exchange-correlation potential is expressed in the generalized gradient approximation using the Perdew–Burke–Ernzerhof (PBE) parametrization.^22^ Bader charge analysis is performed to evaluate the electron density distribution and hence to harvest information about the character of the chemical bond. For this task, the code developed by Henkelmann et al.^23−25^ is used.

**Figure 1:**
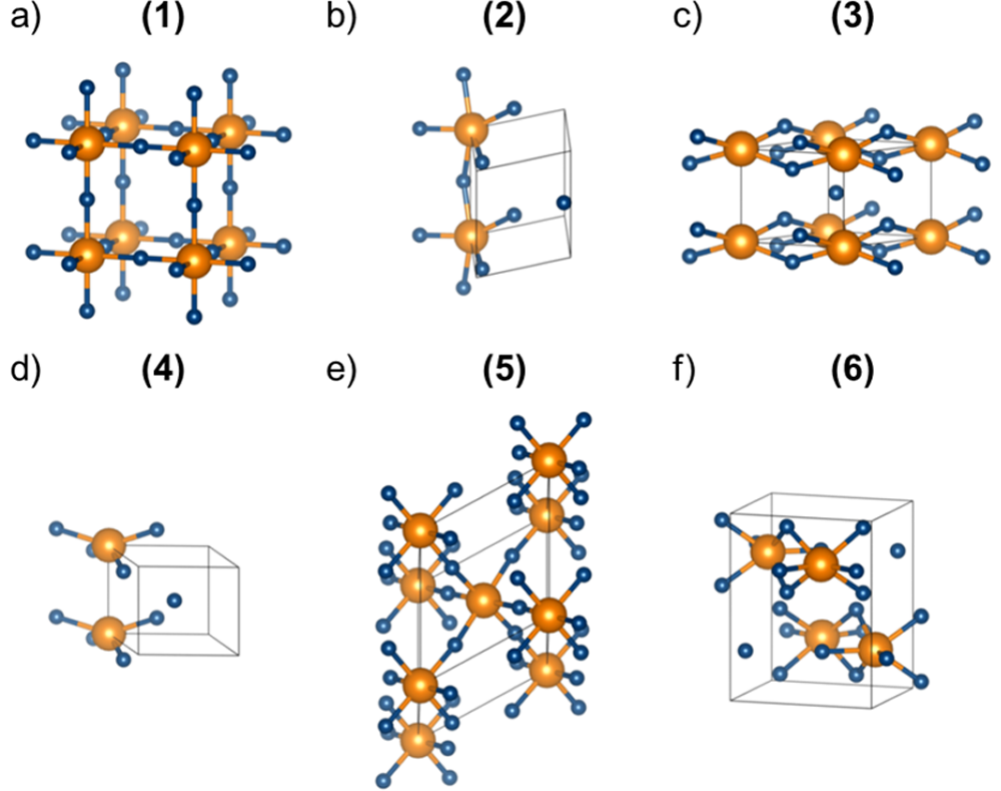
Ball-and-stick representation of the ScF_3_ polymorphs considered in this work: (a) polymorph 1 with a cubic lattice; (b) polymorph 2 with a monoclinic lattice; (c) polymorph 3 with an orthorhombic lattice; (d) polymorph 4 with a monoclinic lattice; (e) polymorph 5 with a trigonal lattice; (f) polymorph 6 with an orthorhombic lattice. Sc atoms are depicted in orange and F atoms in blue. Unit cells are marked by thin black lines; in panel a, the sides overlap with the Sc–F bonds. Plots were produced with the visualization software *VESTA*.^36^

X-ray absorption spectra are calculated from first principles through the solution of the BSE within the all-electron and full-potential framework implemented in the code exciting,^26^ which grants direct access to core electrons as the initial transition states.^27,28^ In practice, the BSE,^29^ which is the equation of motion for the electron–hole correlation function, is solved as an effective, time-independent two-particle Schrödinger equation^27^
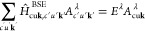
1where *c* and *u* stand for core and unoccupied states, respectively. The Hamiltonian in eq 1 is composed of three terms: *Ĥ*^BSE^ = *Ĥ*^diag^ + *Ĥ*^dir^ + *Ĥ*^x^. The first one, *Ĥ*^diag^, represents vertical transitions from the core to unoccupied levels; the second one, *Ĥ*^dir^, accounts for the electron–hole Coulomb attraction and includes the statically screened Coulomb potential; the third one, *Ĥ*^x^, is the exchange interaction: this term is repulsive due to the opposite charges of the electron and hole. For further details on the BSE Hamiltonian, we refer the readers to the specialized literature.^30−32^ Neglecting the last two terms, namely, solving eq 1 for *Ĥ*^BSE^ = *Ĥ*^diag^, corresponds to the so-called independent-particle approximation (IPA). Eigenvalues and eigenvectors of eq 1 represent excitation energies and excited states, respectively, and both enter the expression of the imaginary part of the macroscopic dielectric function that is commonly adopted to represent absorption spectra:
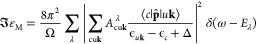
2In the square modulus of eq 2, we recognize the transition matrix elements for the momentum operator **p̂**, where in the denominator a scissors operator Δ is added to mimic the quasi-particle correction to the core and the conduction levels. For a one-to-one comparison of each computed spectrum with the experimental reference, we choose different values of Δ taken with respect to the most intense resonance in the measured energy window. To this end, Δ = 105.8 eV for polymorph 1, Δ = 107.6 eV for 2, Δ = 106.4 eV for 3, Δ = 106.1 eV for 4, Δ = 105.9 eV for 5, and Δ = 102.1 eV for 6 are taken for the Sc K-edge spectra. For the F K-edge spectra, chosen values are Δ = 33.0 eV (1), Δ = 33.8 eV (2), Δ = 33.3 eV (3), Δ = 33.9 eV (4), Δ = 33.1 eV (5), and Δ = 32.5 eV (6). The unit cells of the six considered polymorphs are sketched in Figure 1, and their corresponding crystallographic information is reported in Table 1.

**Table 1:** Space Group, Lattice Name, Formation Energy per Atom (*E*_form_ in eV/atom), and Electronic (*E*_gap_) and Optical (*E*_gap_^opt^) Gaps (Both in eV) Computed for the Considered ScF_3_ Polymorphs

polymorph	space group	lattice	*E*_form_	*E*_gap_	*E*_gap_^opt^
1	*Pm*3̅*m* (No. 221)	cubic	–4.43	6.12	6.64
2	*C*2 (No. 5)	monoclinic	–4.19	5.28	5.52
3	*C*222 (No. 21)	orthorhombic	–3.56	1.84	2.31
4	*C*2 (No. 5)	monoclinic	–3.52	1.80	2.09
5	*R*3̅*c* (No. 167)	trigonal	–4.43	6.14	6.14
6	*Pnma* (No. 62)	orthorhombic	–4.32	5.77	5.81

In the underlying DFT calculations, performed with exciting using the PBE functional, a 4 × 4 × 4 **k** mesh is used to sample the Brillouin zone of all structures except for polymorph 6, for which a 6 × 6 × 6 **k** grid is employed. Muffin-tin radii as large as 1.90 bohr (1 and 2), 1.97 bohr (3), 1.88 bohr (4), 1.92 bohr (5), 1.89 bohr (6) are chosen for Sc atoms, and those as large as 1.71 bohr (1 and 2), 1.77 bohr (3), 1.69 bohr (4), 1.73 bohr (5), 1.69 bohr (6) are chosen for F atoms. The cutoff value *R*_MT_*G*_MAX_ = 8 is adopted for all systems. In the BSE calculations, a Γ-shifted **k** mesh with 4 × 4 × 4 points is employed for all polymorphs except for 3, for which a 6 × 6 × 6 **k** grid is taken. The screened Coulomb potential is computed from the random-phase approximation including 40 (polymorph 1), 30 (polymorph 2), 15 (polymorph 3), 20 (polymorph 4), 60 (polymorph 5), and 90 (polymorph 6) empty bands. Local-field effects are accounted for by choosing energy cutoffs of 108.85 eV (polymorph 1), 81.63 eV (polymorph 2), 163.27 eV (polymorph 3), 108.85 eV (polymorph 4), and 54.42 eV (polymorphs 5 and 6). We include transitions from core levels to conduction bands within an energy range of 37 eV above the conduction-band minimum.

### Experimental Methods

ScF_3_ was provided by the company KBM in the framework of the Horizon 2020 project SCALE. It originates from a Chinese manufacturer and was tested for purity with inductively coupled plasma optical emission spectroscopy and microscopy.^33^ X-ray absorption near-edge structure (XANES) spectra were taken at the PHotons for the Exploration of Nature by Imaging and X-ray absorption fine structure (PHOENIX) undulator beamline of the SLS at PSI, Villigen, Switzerland.

The Sc K-edge spectra were measured at the PHOENIX I branch line, which covers an energy range from 0.8 to 8 keV, using a double-crystal monochromator. To generate monochromatic light at the Sc K-edge, a Si(111) crystal was employed, providing an energy bandwidth for the incoming photons of 0.4–0.5 eV. The powder sample of ScF_3_ was pressed into a pellet, and XANES spectra were taken under vacuum (ca. 10^–5^–10^–6^ mbar) by scanning the energy of the incoming photons over a range from 4400 to 4600 eV and recording the fluorescent light using an energy-dispersive silicon drift detector (four-element Vortex detector, manufacturer Hitachi). The beamsize for the powder sample was 1.5 × 1.5 mm. The absolute flux impinging the sample is on the order of 10^10^–10^11^ photons/s, although not quantitatively measured for each measurement. For normalization of the XANES spectra, the incoming flux, *I*_0_, was taken from a total electron yield (TEY) signal measured on a Ni-coated poly(ethylene terephthalate) foil, located about 1 m upstream of the sample in a vacuum chamber, which was held at ∼10^–7^ mbar. Additionally, high-energy-resolution fluorescence-detected (HERFD) spectra were taken using a new compact von Hamos spectrometer implemented in the end station. Briefly, the spectrometer uses a segmented Si(111) crystal (radius of 7 cm) that is mounted in backscattering geometry. The main axis of the spectrometer is vertically under 90° relative to the incoming beam. The fluorescent photons are collected on a novel 2D Moench detector,^34^ which is mounted on a 2D manipulator to align the in-vacuum spectrometer. The spectrometer is operated with a microfocused beam of 10 × 50 μm. Therefore, despite its small crystal radius, it provides an energy resolution of about 0.5 eV, which is sufficient for emission spectroscopy at tender X-rays. The energy resolution of the spectrometer was derived from the experimentally determined bandwidth of σ ≈ 0.5 eV of elastically scattered photons. This number is a convolution of the true photon bandwidth of 0.3–0.4 eV and a similar spectrometer resolution, both given by the rocking curve of the Si(111) crystal.

For each excitation energy, the Sc Kα fluorescence line is derived from the 2D image taken with the Moench detector. To derive the HERFD spectra, only the central part of the Kα_1_ emission lines is integrated for each excitation energy (see the Supporting Information for further details). The integrated width is about 0.9 eV, which is approximately 3 times the von Hamos spectrometer energy resolution. To obtain the spectra, the excitation energy is chosen with a step of 0.3 eV around the edge, and 0.5 and 3 eV in the pre- and postedge regions, respectively, were chosen.

To measure the X-ray absorption spectra at the F K-edge, the PHOENIX II endstation was used. This endstation is attached to the X-Treme beamline,^35^ which shares the undulator with PHOENIX. Here, monochromatic light is generated using a planar-grating monochromator (energy resolution <0.2 eV). Data were taken in both the TEY and in fluorescence modes using a one-element energy-dispersive silicon drift diode (manufacturer Ketek). The beam was shaped as a round spot of 2 mm diameter. The photon flux on the sample was on the order of 10^10^–10^11^ photons/s, but it was not measured prior to this experiment. All samples were measured in a vacuum chamber kept at about 10^–6^ mbar, which was separated from the beamline vacuum by a 0.5-μm-thin silicon nitride window. The experiments were taken in both the fluorescence and TEY modes. The photon flux, *I*_0_, hitting the sample was measured separately as a TEY signal from a sample-free part of the copper sample holder. This signal was then used to normalize the data. Fundamentally, *I*_0_ could be measured simultaneously by a partially transparent device, such as a Au mesh or a Ni-coated foil, upstream of the sample. At soft energies, such a device would absorb a significant fraction of the photons. This will hamper the *I*_0_ measurement because the photon flux on the *I*_0_ device will differ from that on the sample and because the elemental composition of the device will introduce an unwanted signature to the *I*_0_ measurement. Such pitfalls can be excluded by taking *I*_0_ from a Cu plate inserted into the sample holder. The approach of subsequent *I*_0_ measurement is only possible because the photon source, a third-generation synchrotron, operates in the top-up mode and has sufficient flux stability of less than 1%, which is needed for a proper normalization recorded signal.

## Results and Discussion

### Systems and Structural Properties

To model ScF_3_, we consider the six polymorphs visualized in Figure 1; their structural and electronic characteristics are summarized in Table 1. In addition to the low-temperature, low-pressure cubic crystal 1 and the high-pressure trigonal polymorph 5, two monoclinic phases (2 and 4) and two orthorhombic ones (3 and 6) are considered. To the best of our knowledge, there is no experimental evidence for those structures (2–4 and 6). Yet, it is meaningful to include them in this study because the probed ScF_3_ powder is not assumed to contain monocrystalline particles. In fact, residuals of other polymorphs, although not common, cannot be excluded.

**Figure 2:**
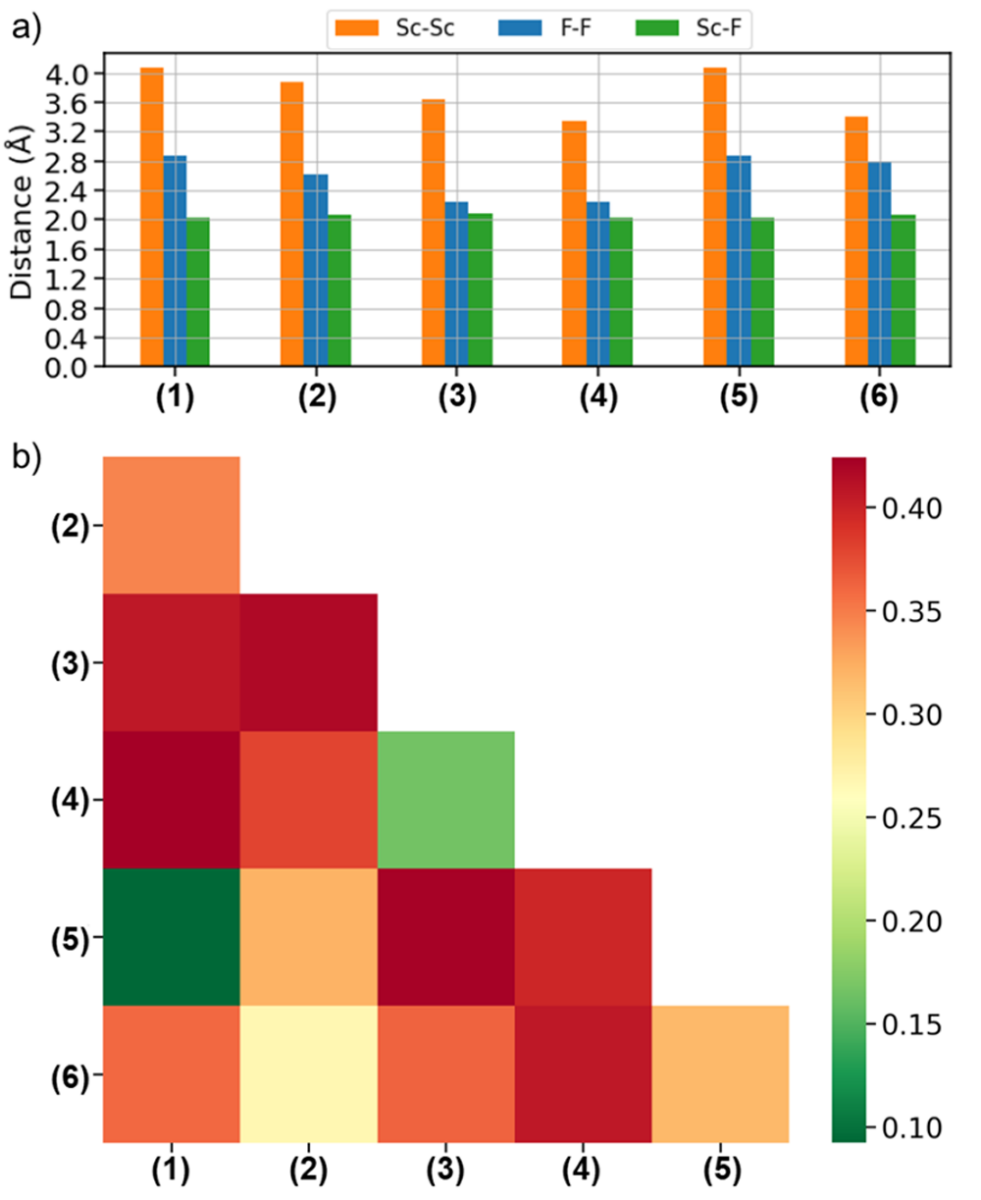
(a) Absolute values of the averaged interatomic distances for the considered ScF_3_ polymorphs and (b) similarity matrix of the averaged interatomic distances based on the pairwise cosine distances evaluated from the F-fingerprint metric: larger similarities correspond to low values (green) and smaller similarities to high values (red).

Quantitative analysis of the structural characteristics of the six considered ScF_3_ polymorphs displayed in Figure 1 reveals significant similarities among them. First, we examine the mutual distances between Sc and F that are evaluated as the positions of the first peak in the F-fingerprint function^37^ of the element pair (Figure 2a). Interestingly, we find that structures 1 and 5, namely, the experimentally resolved low- and high-temperature phases of ScF_3_, respectively, are characterized by almost identical interatomic distances, in line with the understanding that the pressure-induced phase transition corresponds to a rigid rotation of the octahedra in the lattice.^10^ The separation between pairs of Sc (F) atoms amounts to 4.1 Å (2.9 Å), while the Sc–F bond length is equal to 2.0 Å. The structural similarity between these two phases is also in line with the almost identical X-ray diffraction spectra of the two polymorphs (Figure S4). In the other polymorphs, this distance does not change significantly: this behavior can be understood considering the Sc–F bond as an intrinsic property of the compound, regardless of the lattice arrangement. On the other hand, both F–F and Sc–Sc distances undergo a reduction in the structures that have not been experimentally resolved yet (Figure 2a). The smallest values are found in the monoclinic phase 4, where the Sc–Sc (F–F) separation becomes equal to 3.3 Å (2.2 Å). These reductions are not unexpected given the relatively small unit-cell volume of this phase (Figure 1 and Table S1). In the orthorhombic polymorph 3, a similar value of the F–F separation (∼2.2 Å) is accompanied by a slightly larger Sc–Sc distance (3.6 Å). In contrast, in the other orthorhombic phase 6, the interatomic separations Sc–Sc and F–F are the smallest, being 3.4 and 2.8 Å, respectively. Finally, in the monoclinic structure 2, the Sc–Sc and F–F distances are equal to 3.9 and 2.6 Å, respectively.

A more comprehensive interpretation of the interatomic distances in the considered ScF_3_ polymorphs can be obtained by adopting the F-fingerprint metric developed by Oganov and Valle.^37^ By discretization of the sum of the radial distribution function of each element pair, an *n*-dimensional vector is obtained, where *n* is the number of discretized bins, and a quantitative similarity can be defined through the cosine distance
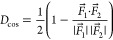
3where *F⃗*_1_ and *F⃗*_2_ are the radial distribution vectors of two different crystals. Small values of *D*_cos_ indicate close similarity (Figure 2b), where the results of eq 3 are plotted in a color matrix by contrasting each pair of structures among the six considered ScF_3_ polymorphs. The similarity between the experimental low- and high-pressure phases (1 and 5) is evident from the plot. Moreover, the resemblance between crystals 3 and 4 is also highlighted. The remaining two polymorphs, 2 and 6, are structurally closer to each other than to any other structure. On the other hand, their similarity is less pronounced than that for the other two pairs of crystals. Overall, it is noteworthy that the computationally predicted phases exhibit more remarkable structural differences among each other than the two experimentally resolved polymorphs.

### Energetic Stability

In the next step of our analysis, we assess the stability of the considered ScF_3_ crystal structures by calculating their formation energy per atom (*E*_form_) according to the formula
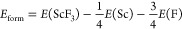
4where *E*(*x*) is the total energy per atom of compound *x*. For the elemental phases of F and Sc, the most stable experimentally crystal structure available in Materials Project^38,39^ has been taken. We emphasize that the total energies entering eq 4 are computed from DFT, assuming the system to be in a fixed geometry at 0 K. As such, no thermodynamic effects are taken into account, and the obtained values provide only a qualitative trend of the relative stability of the polymorphs.

The results shown in Table 1 are consistent with the structural similarities discussed above. The most stable structures are the low- and high-temperature polymorphs (1 and 5, respectively). Interestingly, our calculations yield identical formation energies for these two materials. Structures 3 and 4 exhibit very similar values of *E*_form_, differing from each other by only 40 meV/atom but being significantly less negative (by about 600 meV/atom) than those of polymorphs 1 and 5. In contrast, structures 2 and 6 are characterized by formation energies equal to −4.19 and −4.32 eV/atom, respectively.

We remark that this analysis holds for single crystals modeled on the ideal structures sketched in Figure 1. It is expected that the presence of defects, such as interstitial atoms and vacancies, will alter this picture. A corresponding analysis is, however, beyond the scope of the present work.

### Charge Distribution

**Figure 3:**
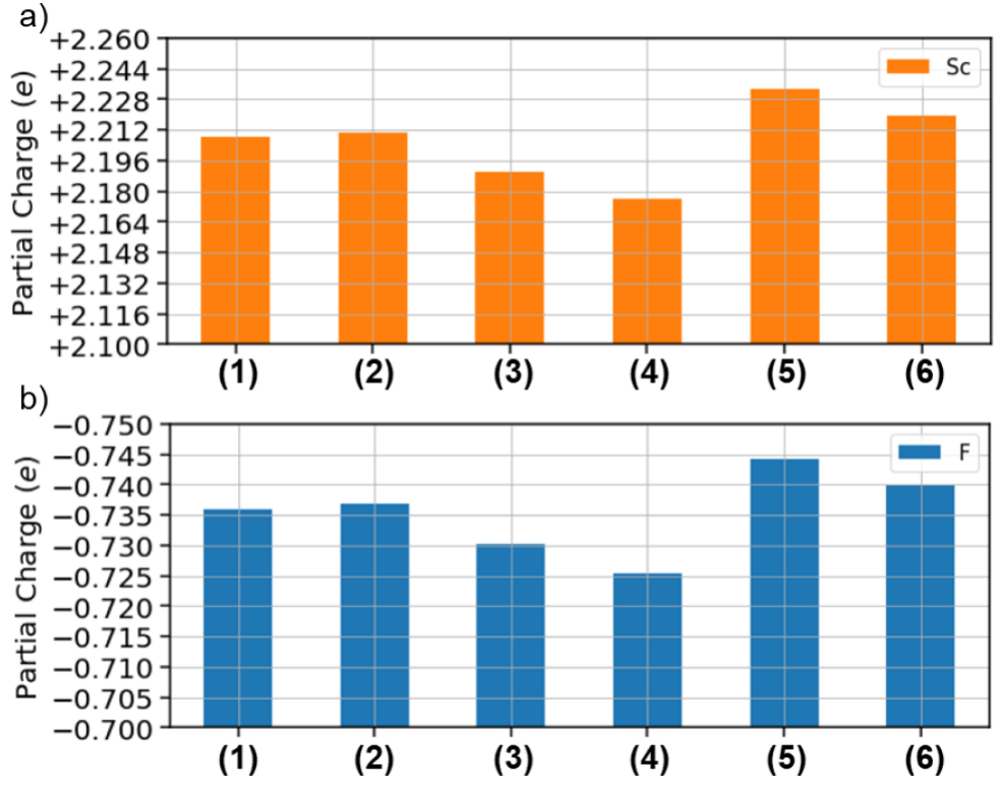
Mean partial charges calculated with the Bader scheme of (a) Sc and (b) F atoms in the six considered ScF_3_ polymorphs.

To analyze the chemical bonds in the different polymorphs, we performed partial charge analysis using the Bader scheme.^23^ In agreement with the knowledge that ScF_3_ is an ionic crystal, in all considered phases, the Sc atom is positively charged and the F atoms are negatively charged (Figure 3). Yet, the relative variations of the partial charges in the different polymorphs provide valuable information about the nature of the bonds: the larger the absolute values of the charges on each atomic species, the more ionic the bond. In the experimental high-pressure ScF_3_ polymorph 5, the positive charge on Sc is about 2.33 e and the negative charge on each F atom is almost as high as −0.75 e, thereby pointing to a high degree of ionicity in the bonds of this phase. In contrast, the least pronounced ionicity among the considered polymorphs is found in the monoclinic crystal 4, where the charge on Sc (F) amounts to 2.17 e (−0.72 e). The other considered structures exhibit intermediate behaviors, with the orthorhombic crystal 6 having an Sc–F bond that is almost as ionic as that in phase 5 and polymorph 3 displaying a more covalent bond similar to 4.

From this analysis, it is evident that the similarities among the polymorphs based on interatomic distances and formation energies do not affect the partial charges. However, it should be noticed that, in absolute values, variations on the partial charges are small, ranging within an interval of less than 0.1 e. This small spread is actually compatible with the negligible variations of the Sc–F distances among the polymorphs (Figure 2a).

### Electronic Properties

In the next step of this analysis, we inspect their electronic properties, focusing on the band gaps and on the density of states (DOS). With the exception of the high-pressure experimental polymorph 5, which exhibits a direct band gap at Γ (Table 1), all of the other structures are characterized by an indirect band gap (Figure S2). The largest gaps are found for the known low- and high-pressure polymorphs (1 and 5) where *E*_gap_ = 6.12 eV and 6.14 eV, respectively. The smallest direct gap obtained from DFT—hereafter named the *optical* gap—in the low-pressure polymorph 1 is equal to 6.64 eV. Structures 2 and 6 are also wide-band-gap insulators with fundamental gaps as high as 5.28 and 5.77 eV, respectively, while the optical gaps amount to 5.52 and 5.81 eV, respectively (Table 1). Conversely, structures 3 and 4 are characterized by much smaller band gaps, around 2 eV. Such a drastic reduction of the band-gap size in these polymorphs is a signature of their lower stability with respect to the experimental ones, as confirmed also by the values of the formation energies reported in Table 1. Overall, the values of the band gaps, both indirect and direct, follow trends analogous to those found for the interatomic distances (Figure 2b) and formation energies (Table 1).

**Figure 4:**
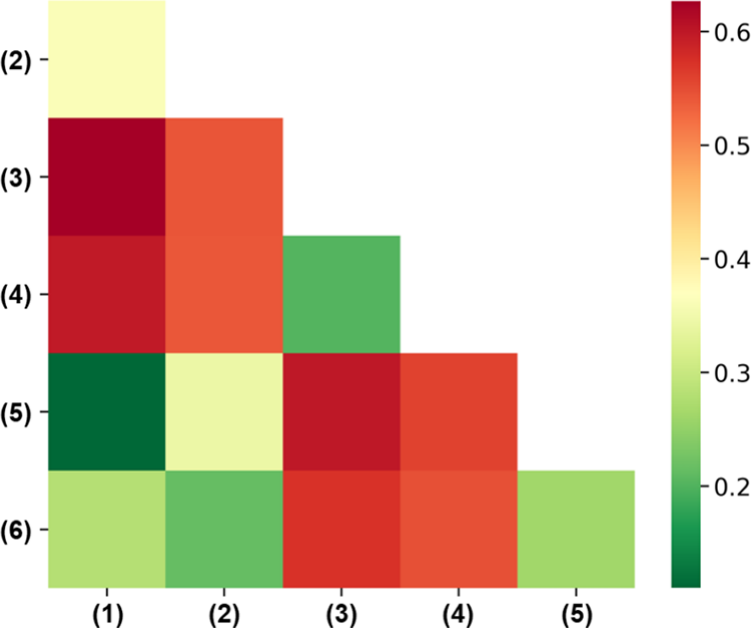
Similarity matrix based on the pairwise cosine distances evaluated from the F-fingerprint metric for the DOSs computed for the six considered ScF_3_ polymorphs: Larger similarities correspond to low values (green) and smaller similarities to high values (red).

The similarity among the considered polymorphs can be additionally evaluated for their DOSs within an energy range between −15 and +15 eV around the Fermi energy. The DOSs for the six polymorphs are reported in Figure S3. Here, we inspect the similarity matrix obtained by applying to these results the F-fingerprint metric.^37^ In this case, some preprocessing of the data is necessary to evaluate eq 3. First, all DOSs are normalized with respect to the number of atoms in the unit cell and referenced to the energy value of the valence-band maximum. Then, the pairwise distance of the discretized functions is calculated, and the cosine distance is obtained. The results plotted in Figure 4 confirm the similarities between the pairs of structures seen in the structural analysis and in the formation energies: polymorphs 1 and 5 exhibit similar characteristics in the DOS; the same is true for structures 3 and 4 as well as structures 2 and 6. Interestingly, the DOSs of the two polymorphs 2 and 6 are also quite similar to those of structures 1 and 5. The electronic structures of 2 and 3, instead, are quite different from those of all other polymorphs.

### X-ray Spectroscopy

In the second part of our study, we discuss the X-ray absorption spectra of ScF_3_ at the Sc and F K-edges.^40^ The results of first-principles calculations are reported on the six considered polymorphs, as computed from the solution of the BSE (eq 1) as well as in the IPA. By contrasting against each other the results obtained with these two methods, we can assess the role of excitonic effects, including quantifying exciton binding energies^41,42^ and connecting the spectral fingerprints to the electronic structure of the materials. A comparison with the HERFD experimental data enables identification of the spectral fingerprints and assessment of the composition of the sample in terms of the considered polymorphs.

### Sc K-Edge Spectra

**Figure 5:**
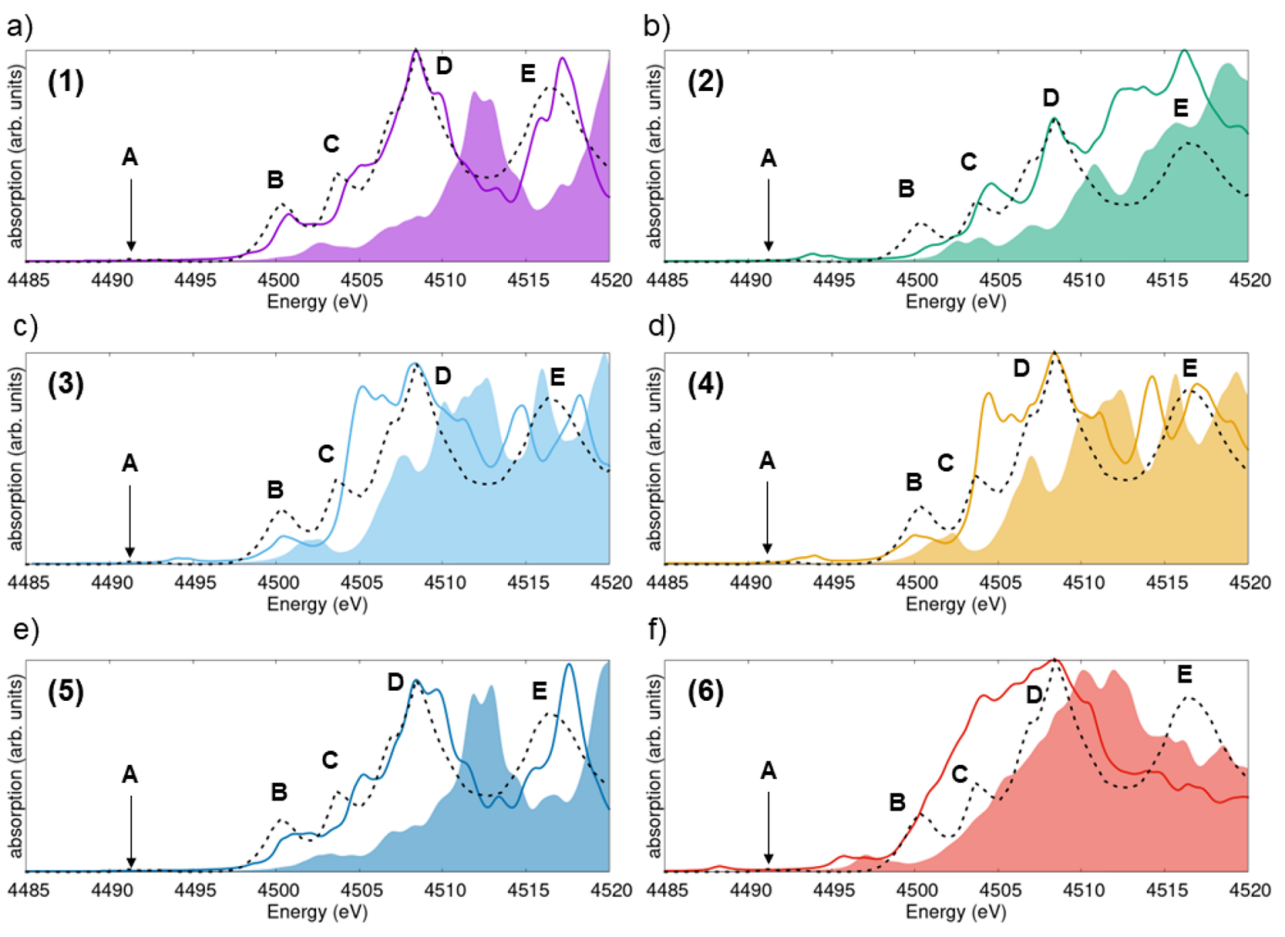
X-ray absorption spectra from the Sc K-edge calculated for polymorphs (a) 1, (b) 2, (c) 3, (d) 4, (e) 5, and (f) 6. Solid lines (shaded areas) indicate results, including (excluding) electron–hole interactions, as computed from the solution of the BSE (in the IPA) with a Lorentzian broadening on 0.5 eV. The HERFD result (dashed line) is overlaid with all calculations, and the main features therein are labeled in capital letters. The BSE spectra are aligned to the experimental reference with respect to the energy peak D.

We start from the X-ray absorption spectrum of ScF_3_ taken at the Sc K-edge (Figure 5). The results calculated for each considered polymorph are reported on each panel and overlaid with the measurement (dashed line). First, let us consider the computational results obtained for the different structures (BSE, solid line; IPA, shaded area). All simulated spectra exhibit the typical shape from the K-edge of transition metals:^43^ a weak absorption onset is followed by stronger resonances at increasing energies. Some phases are characterized by a prepeak: this is found around 4493 eV in polymorphs 2–4, while in structure 6, it appears at approximately 4487 eV. Notably, such a feature is absent in the calculated spectra of polymorphs 1 and 5, representing the low- and high-pressure single-crystalline phases, respectively. A comparison among the computational results reveals similarities to those highlighted in the structural properties, energetic stability, and charge distribution analysis reported above. Specifically, the spectra obtained for phases 3 and 4 resemble each other closely. Likewise, the spectra of polymorphs 1 and 5 are characterized by similar features.

It is worth a reminder that the spectra discussed above are computed from the explicit evaluation of the matrix elements between the core and conduction states (see eq 2). Information about the chemical environment of the excited atoms is intrinsically encoded in the electronic structure obtained from DFT, and further analysis on the local geometry of the targeted species is not requested by the adopted approach. For further details and comparisons among different methods for X-ray spectroscopy, we refer the readers to specialized reviews.^44,45^

Excitonic effects are sizable in all examined structures and manifest themselves mainly through redshifts of the peaks toward lower energies without a significant redistribution of the spectral weight among the absorption maxima. The spectra of phases 3 and 4, however, do not exhibit this behavior, especially in the region between 4500 and 4515 eV. Exciton binding energies, evaluated as the difference between excitation energies in the BSE solution and in the IPA,^27,41,42,46^ are quite large in these spectra, on the order of a few electronvolts and reaching values of approximately 5 eV for the most intense maxima in the spectra of polymorphs 1 and 5. This result is not surprising considering the ionic character of ScF_3_ and the consequent low screening therein. Similar values were obtained for core-level excitations in organic materials.^41^

**Figure 6:**
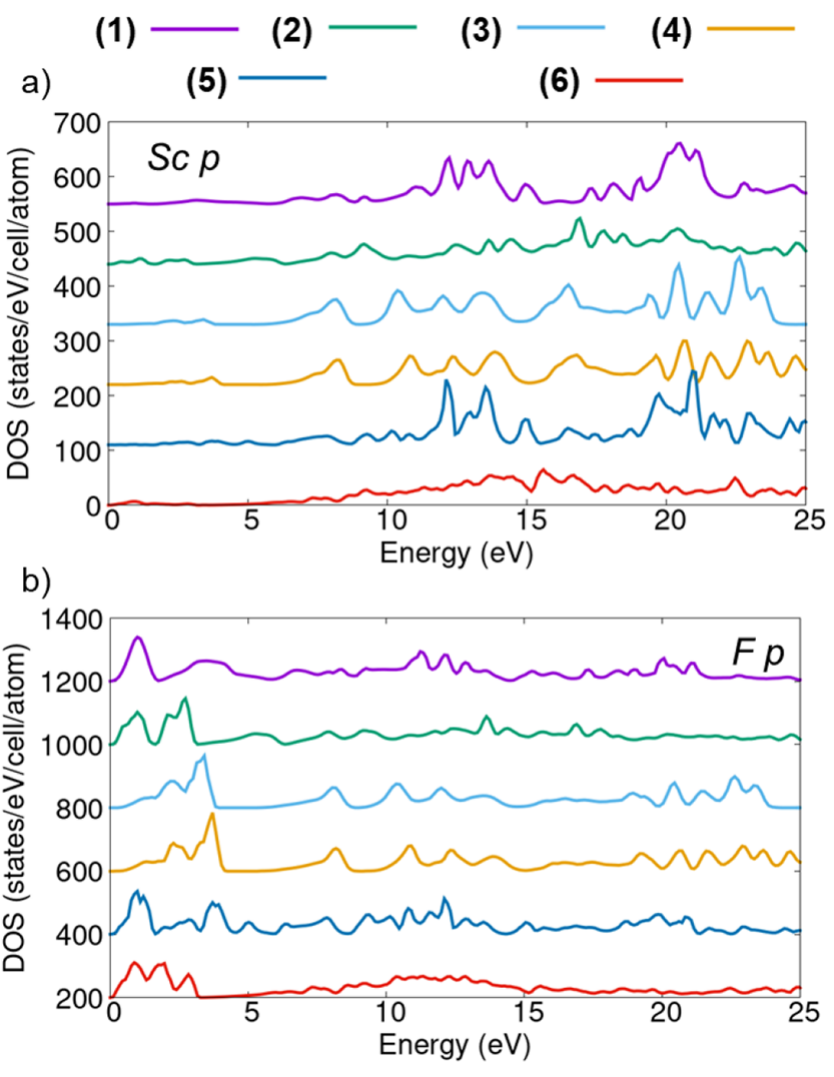
(a) Sc and (b) F contributions to the p states in the projected DOS of the six ScF_3_ polymorphs considered in this work. The conduction-band minimum is set at 0 eV.

The spectra computed in the IPA (Figure 5, shaded areas) can be closely connected with the Sc p-orbital contributions to the unoccupied partial DOSs (PDOSs; Figure 6a), which represent the target states of these transitions. The overall similarity between the BSE and IPA spectra, except for the shifts of the resonances related to the excitonic effects discussed above, eases this analysis. The strong resonances in the spectra around 10 eV above the (weak) onset are reflected in the PDOSs, where corresponding peaks are particularly pronounced in the energy region comprised between 10 and 15 eV (Figure 6a). The similarities between the spectra of these low- and high-temperature polymorphs (1 and 5, respectively) are reflected also in their PDOSs, especially between 10 and 15 eV. The maxima around 20 eV differ more substantially in these two structures. Similarities are evident also between the Sc p-orbital contributions in the PDOSs of phases 3 and 4 and, to a lesser extent, due to the overall lower DOS magnitude also for structures 2 and 6. Careful inspection of Figure 6a reveals the presence of weak states from the conduction band edge up to 5 eV in the PDOSs of structures 2–4 and 6. These states give rise to the prepeak in the spectra of these polymorphs. In contrast, no contributions from the available Sc p states are seen in the PDOSs of the low- and high-temperature phases 1 and 5, in agreement with the absence of any corresponding prepeaks in their spectra (Figure 5).

With the knowledge gained from this analysis, we can finally proceed with a comparison between the calculated spectra and the experimental data (dashed lines in Figure 5). The measured spectrum is corrected for overabsorption; see details in Figure S6. Five main features are identified in the measurement: the weak prepeak A, three maxima of increasing intensity building up the onset (B–D), and the high-energy peak E. The prepeak A can be associated with the low-energy feature in the spectra computed for polymorphs 2–4 and 6, although in all of these cases, the relative energy of this weak maximum is shifted with respect to the experimental reference. Notice that the computational results are aligned with the energy of the most intense peak D. The peaks at the onset, B–D, can be identified in all BSE spectra, although the relative energies and oscillator strengths vary, thereby affecting the agreement with the experiment. The spectra calculated for polymorph 1 best reproduce all of these characteristics (Figure 5a), including the relative oscillator strengths of the main peaks. Good agreement with the experiment can be claimed also by the BSE result obtained for phase 5, where, in particular, the substructures of peak D are better captured than those in the spectrum of phase 1. In the spectra computed for the other structures, we notice both an energy misalignment of the peaks and an incorrect description of their relative intensities. All in all these findings suggest that the sample on which the HERFD measurement shown in Figure 5 was taken contains predominantly phase 1 possibly mixed with polymorph 5. The missing prepeak A in these two phases may point to the presence of additional coexisting polymorphs where this feature is visible. However, it can also be associated with defects or sample impurities that cannot be resolved by the calculations.

### F K-Edge Spectra

**Figure 7:**
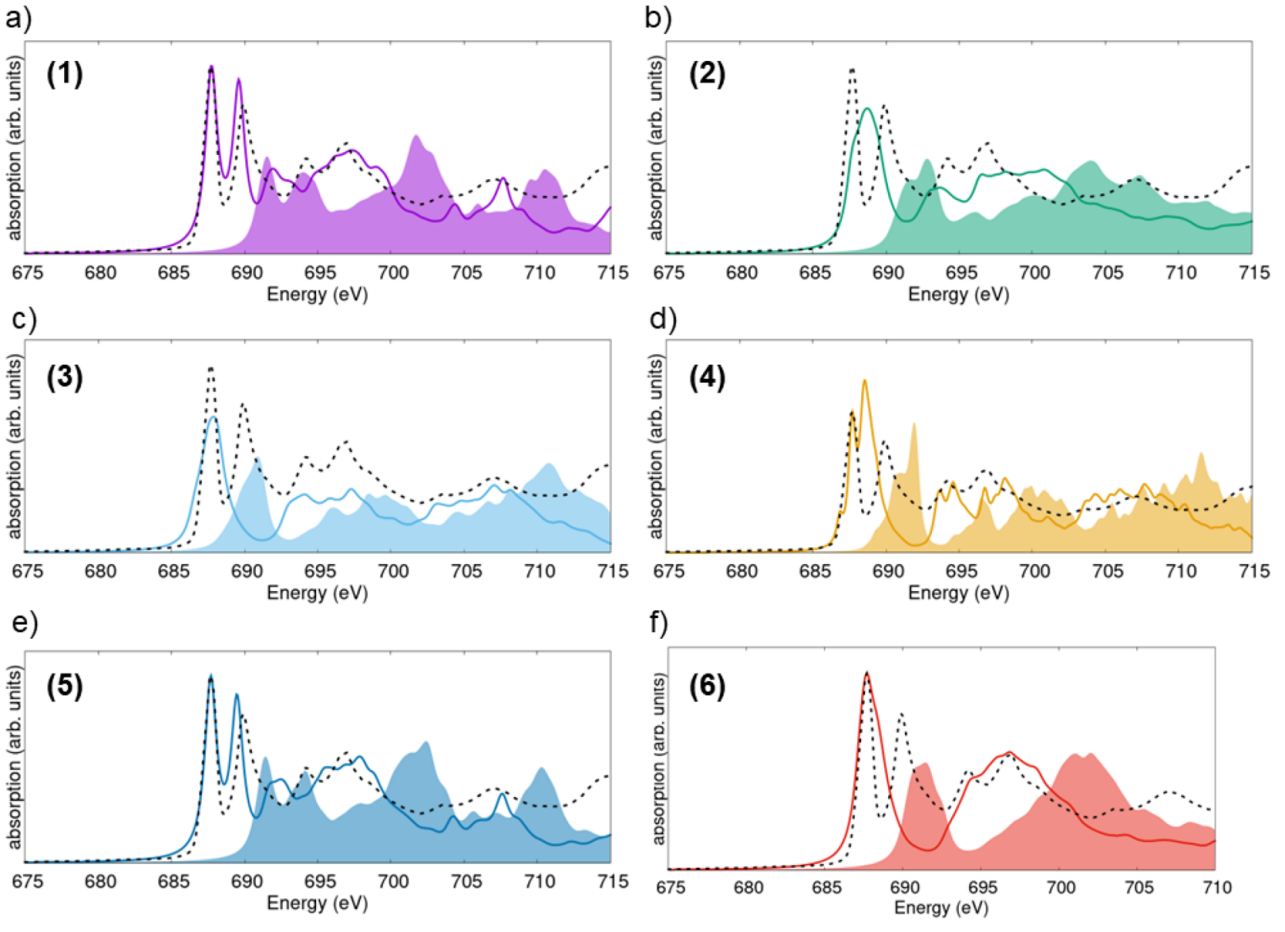
X-ray absorption spectra computed from the F K-edge of ScF_3_ in polymorphs (a) 1, (b) 2, (c) 3, (d) 4, (e) 5, and (f) 6. Solid lines (shaded areas) indicate results, including (excluding) electron–hole interactions, as computed from the solution of the BSE (in the IPA). A Lorentzian broadening on 0.5 eV is applied to all spectra. The HERFD result (dashed line) is overlaid with all calculated spectra. The BSE spectra are aligned to the experimental reference at the energy of the peak at 687 eV.

Next, we examine the X-ray absorption spectra obtained from the F K-edge (Figure 7). The results obtained for polymorphs 1 and 5, polymorphs 3 and 4, and polymorphs 2 and 6 exhibit mutually similar features. In the spectra of structures 1 and 5, which correspond to the low- and high-temperature phases of ScF_3_, two sharp maxima dominate the onset. The presence of two bright excitations at the lowest energies is visible also in the spectra of phase 4, while in the spectra of polymorphs 2, 3, and 6, their energy separation is within the chosen broadening, and, therefore, only one peak with a profile slightly deviating from an ideal Lorentzian is seen in Figure 7b,c,f.

Excitonic effects, evaluated again through a comparison between the BSE and IPA results, are evident in all spectra. In contrast to the Sc K-edge spectra where the explicit inclusion of electron–hole correlations in the calculations leads to an overall shift of the spectral weight toward lower energies and only to a slight redistribution of the oscillator strength (Figure 5), here it enables the correct description of the sharp lowest-energy resonances (Figure 7). In fact, although these peaks are present also in the IPA solution, their intensity is drastically underestimated due to the missing electron–hole interaction. This is a well-known shortcoming of this approximation.^42,46−49^ Quantitatively, exciton binding energies estimated for the F K-edge spectra of the considered ScF_3_ polymorphs are on the order of 3–4 eV, i.e., systematically larger than those obtained from the Sc K-edge.

The inspection of the PDOS shown in Figure 6b helps to explain the dominant spectral weight at low energies in these spectra. For all considered systems, a pronounced peak associated with the available F p orbitals at the bottom of the conduction band is clearly visible for polymorphs 1 and 5. In the PDOS computed for structures 2 and 6, available F p states are spread over a broader energy window. Finally, in the PDOS of polymorphs 3 and 4, the maxima for F p orbitals are shifted at higher energies, between 2.5 and 4.5 eV. The presence of F p states at low energies in the unoccupied region follows chemical intuition: the empty orbitals of the 2p shell of F lie low in energy due to the tendency of this element to accept electrons to complete the octet. The PDOS contributions displayed in Figure 6b support the similarity between the IPA spectra of polymorphs 1 and 5 as well as of phases 3 and 4. Notably, for the latter two compounds, excitonic effects act differently, giving rise to a two-peak structure at the onset in 4 and to a single-peak structure in 3 in the BSE solutions. In contrast, the similarity between the spectra of the experimentally stable structures 1 and 5 is reflected in both the IPA and the BSE solutions.

The experimental data are very well reproduced by the results computed for polymorphs 1 and 5 (Figure 7a,e). The two sharp resonances at the onset feature relative energies and intensities in close agreement with the measurements (notice that the computational results have been aligned in energy and intensity to the experimental ones with respect to the lowest-energy peak). Higher-energy maxima are also correctly reproduced. The absence of the double-peak structure in the BSE spectra of the other polymorphs hinders the agreement with the experiment in this region. However, especially for phase 3 and partly for phase 4, the higher-energy region (693–710 eV) is correctly reproduced by the calculations. Analysis of the F K-edge spectra confirms the clear prevalence of polymorphs 1 and 5 in the measured sample, as anticipated from the study of the Sc K-edge spectra (Figure 5). However, also from this analysis, the presence of other polymorphs in the probed sample cannot be totally excluded. In particular, the excellent agreement between the experimental spectral profile in the region 693–710 eV featured by the spectrum calculated for phase 3—even superior to the results obtained for polymorphs 1 and 5 in this energy range—may suggest residues of this phase in the sample. On the other hand, the presence of defects and impurities could also be responsible for these effects. Addressing this topic goes, however, beyond the scope of the present work.

## Summary and Conclusions

In summary, we have performed a systematic first-principles analysis of six computationally predicted ScF_3_ polymorphs by assessing their energetic stability, charge-density distribution, and electronic properties, in order to identify similarities between the various phases. Our results have revealed the analogy between the two experimentally resolved low- and high-temperature phases, indirectly confirming that the transition between them consists of a rigid lattice rotation. Analysis of the X-ray absorption spectra of all structures sheds light on the electronic origin of the resonances and on the prominent excitonic effects characterizing them. A comparison against the HERFD data reveals very good agreement with the results computed for the low- and high-temperature phases, confirming their predominance in the samples. Some slight differences between the computed spectra of these systems and the experimental data raise some questions regarding the residual presence of other polymorphs, to date only computationally predicted, in the measured powder samples. Yet, only a detailed analysis of the influence of defects and impurities on the spectra can support this hypothesis, ruling out that such discrepancies are caused by these more common factors.

In conclusion, this work provides new insight into the structure–property relationships of ScF_3_ and its polymorphs, including both the experimentally resolved phases and the computationally predicted ones. Specifically, the information provided by the *ab initio* characterization of the energetic stability, charge distribution, and electronic structure extends the existing knowledge of these systems. Further insight is given by analysis of the X-ray absorption spectra by contrasting state-of-the-art measurements and calculations. We are confident that the results obtained for the computational phases will stimulate further experimental investigations on ScF_3_ single crystals.

## Supporting Information Available

Crystallographic information about the simulated polymorphs, additional computational results (similarity matrices for the partial charges, band structure, and DOS of the considered polymorphs), and additional experimental data (X-ray diffraction patterns and resonant inelastic X-ray scattering map at the Sc K-edge) (PDF)
