# Supplementary material for: Electronic Structure and Core Spectroscopy of Scandium Fluoride Polymorphs
Source: Inorg Chem. 2023 Mar 1;62(10):4238–47. doi: 10.1021/acs.inorgchem.2c04357 (PMC10015455; doi:10.1021/acs.inorgchem.2c04357)
Supplement: Supplementary file 1 — ic2c04357_si_001.pdf [file ic2c04357_si_001.pdf]

SUPPORTING INFORMATION

Electronic Structure and Core Spectroscopy of Scandium Fluoride Polymorphs

Fabiana Machado Ferreira De Araujo,[†,‖] Daniel Duarte-Ruiz,[†,‖] Holger-Dietrich Saßnick,[†] Marie C. Gentzmann,[‡,⊥] Thomas Huthwelker,[¶] and Caterina Cocchi[*,†,§]

†*Institute of Physics, Carl-von-Ossietzky Universität Oldenburg, 26129 Oldenburg, Germany*

‡*Bundesanstalt für Materialforschung und –prüfung (BAM), Unter den Eichen 87, 12205, Berlin, Germany*

¶*Paul Scherrer Institut, Swiss Light Source (SLS), 5232, Villigen, Switzerland*

§*Physics Department and IRIS Adlershof, Humboldt-Universität zu Berlin, 12489 Berlin, Germany*

‖*Contributed equally to this work*

⊥*Present address: Bundesanstalt für Geowissenschaften und Rohstoffe (BGR), Stilleweg 2, 30655, Hannover, Germany*

E-mail: caterina.cocchi@uni-oldenburg.de

Additional Computational Results

Table S1: Lattice parameters (vectors and angles), unit cell volume, and Materials Project[1] ID of the six considered ScF_3 polymorphs.

Polymorph	a (Å)	b (Å)	c (Å)	α	β	γ	V (Å^3)	Materials Project ID
(1)	4.07	4.07	4.07	90°	90°	90°	67.40	mp-10694
(2)	4.62	6.21	4.07	90°	114.8°	90°	53.02	mp-1179500
(3)	3.64	7.04	3.44	90°	90°	90°	44.06	mp-695817
(4)	3.69	7.05	3.35	90°	95.33°	90°	43.37	mp-634805
(5)	5.74	5.74	5.74	60.09°	60.09°	60.09°	133.98	mp-1078300
(6)	4.82	5.47	6.97	90°	90°	90°	183.53	mp-1094067

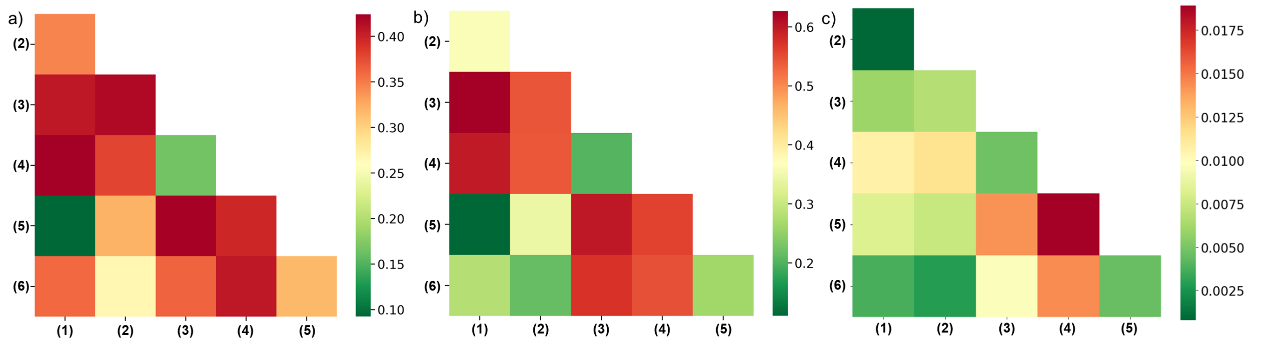

Figure S1: Similarity matrices calculated via the F-fingerprint metric of a) the crystal structures, b) electronic density of states (DOS), and c) absolute mean difference of the partial charges. While the matrices based on the crystalline structures and electronic DOS show qualitatively the same features (cf. panels a and b), the matrix based on partial charges (panel c) only partly matches with the previous ones. This indicates that the partial charges do not provide a reliable metric to assess the similarity of structural and electronic properties of different polymorphs. More complex descriptors based on the real space electronic density, on the other hand, are able to resolve the similarity of the structures better.

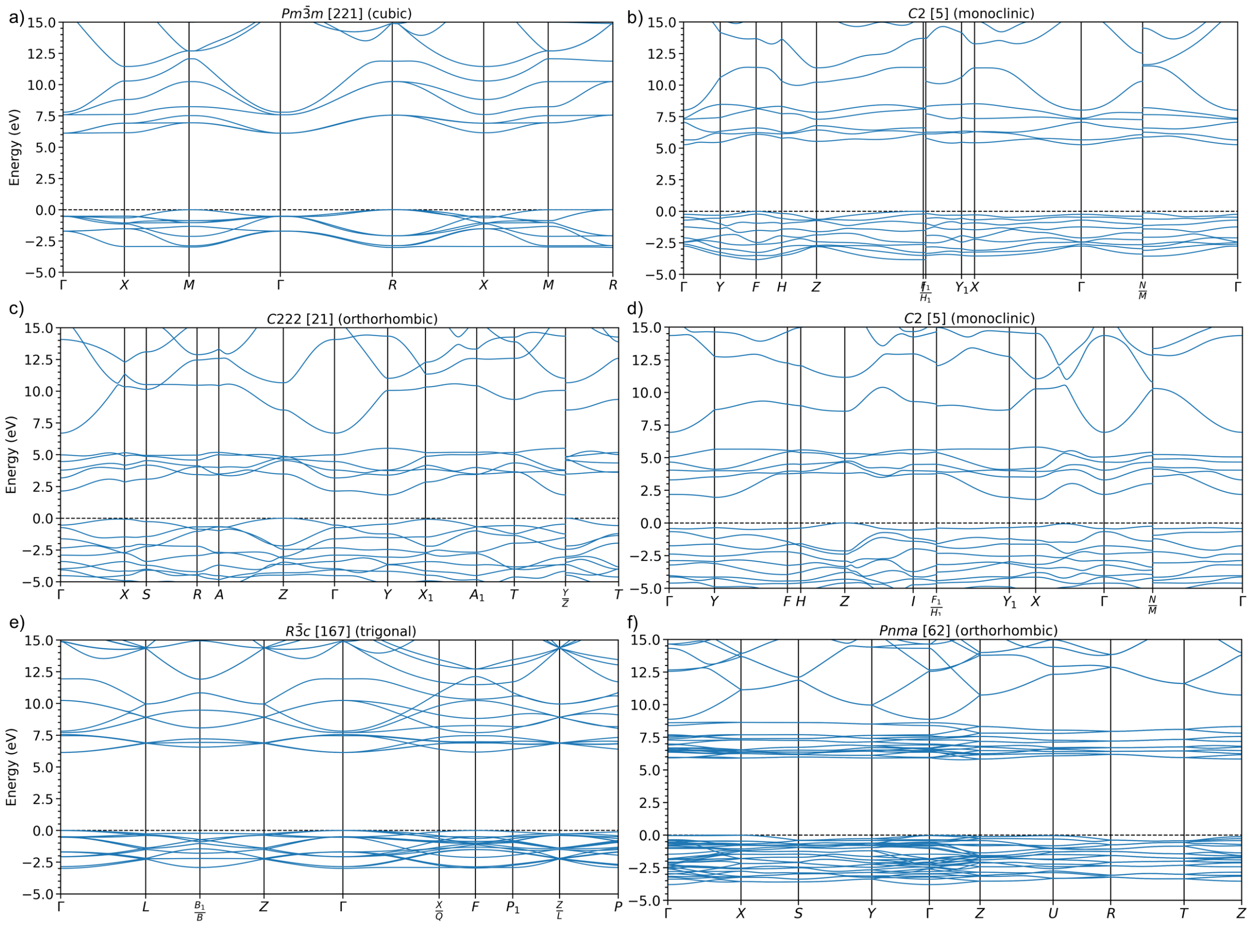

Figure S2: Band structures of ScF_3 in a) polymorph (1), b) polymorph (2), c) polymorph (3), d) polymorph (4), e) polymorph (5), and f) polymorph (6). The energy is set to zero at the valence band maximum (dashed line).

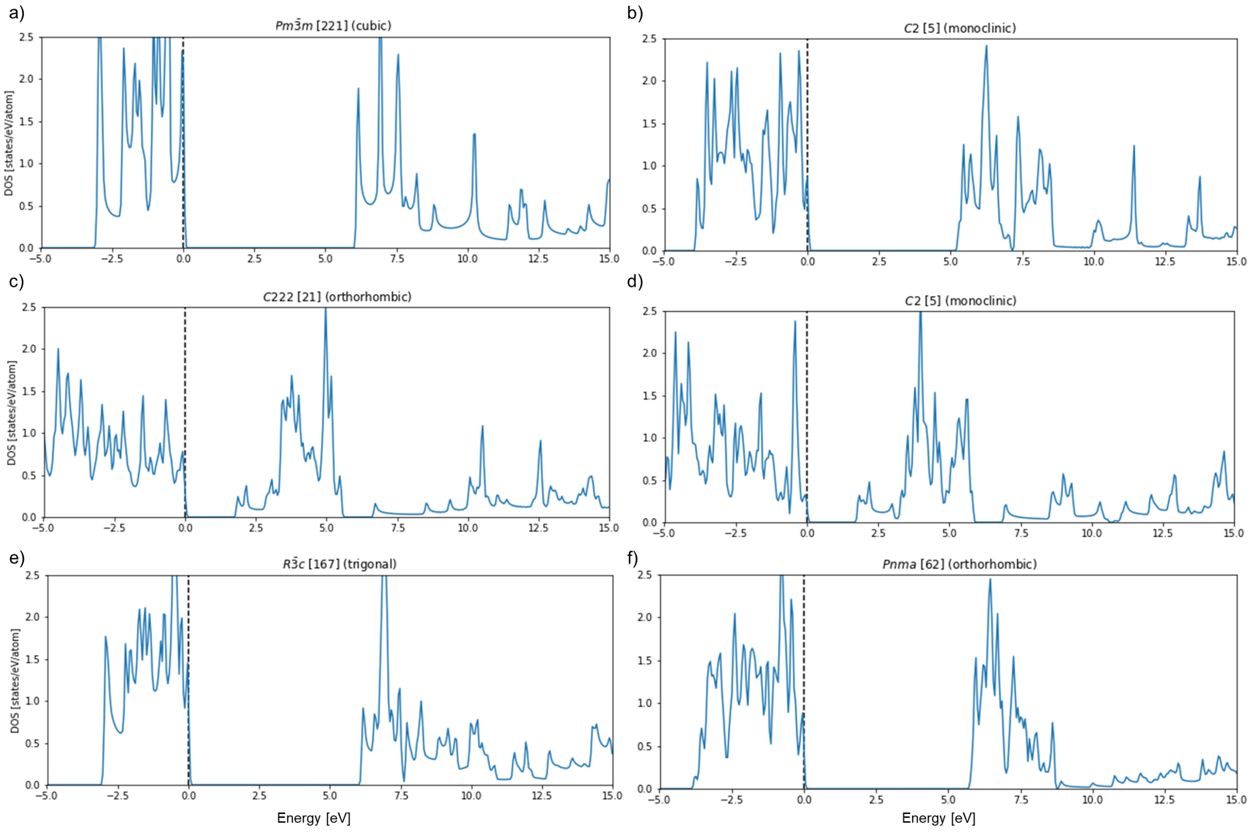

Figure S3: Density of states (DOS) of ScF_3 in a) polymorph (1), b) polymorph (2), c) polymorph (3), d) polymorph (4), e) polymorph (5), and f) polymorph (6). The energy is set to zero at the valence band maximum (dashed line).

Additional Experimental Results

X-Ray Diffraction

X-ray diffraction (XDR) data recorded for the ScF_3 sample analyzed in this work are compared against references for the cubic and the trigonal phase taken from the ICDD Powder Diffraction File-Database, see Figure S4. Due to the stark similarity between the patterns of the two experimentally known polymorphs, this technique provides too little information to uniquely determine the crystallographic structure of the power sample.

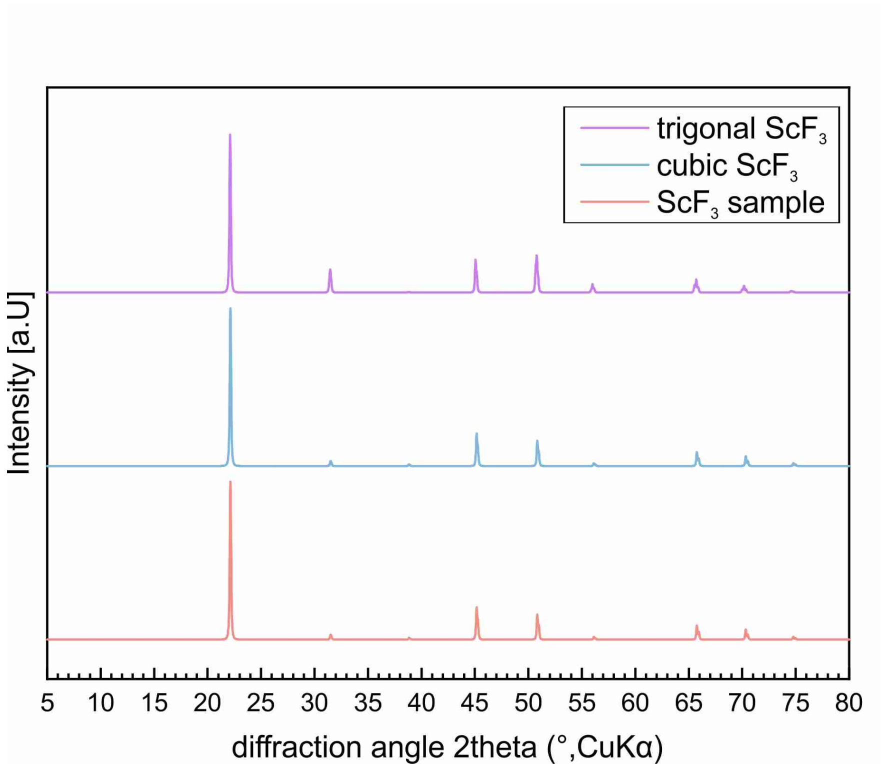

Figure S4: X-ray diffractogram of the ScF_3 sample and of the two experimentally known structures, cubic and trigonal, taken from the ICDD Powder Diffraction File-Database. (PDF Entry cubic: 00-046-1243, PDF Entry trigonal: 01-085-1078).

X-Ray Absorption Spectroscopy

The HERFD spectra were derived from the full RIXS plane, which is shown in Figure S5, left panel. The RIXS plane shows the high-resolution emission spectrum (y-axis) as a function of the excitation energy. Both the Sc $K_{\alpha 1}$ (at 4079.5 eV) and $K_{\alpha 2}$ (4074.5 eV) emission lines are shown. The energy calibration of the emission spectrometer is based on the elastic scattering lines taken from the sample in a separate measurement. The figure shows the counts in a logarithmic scale to visualize also the pre-edge peaks at 4491 and 4492 eV. For total fluorescence yield XAS, for each excitation energy, the full emission spectrum was averaged, which is equivalent to recording the emission line using an SDD detector with an energy resolution of about 150 eV. For the HERFD XANES, only the small central part of the emission line is integrated. Here the range 4079.5±0.45 eV is used, as indicated by the two black lines on the right panel of Figure S5.

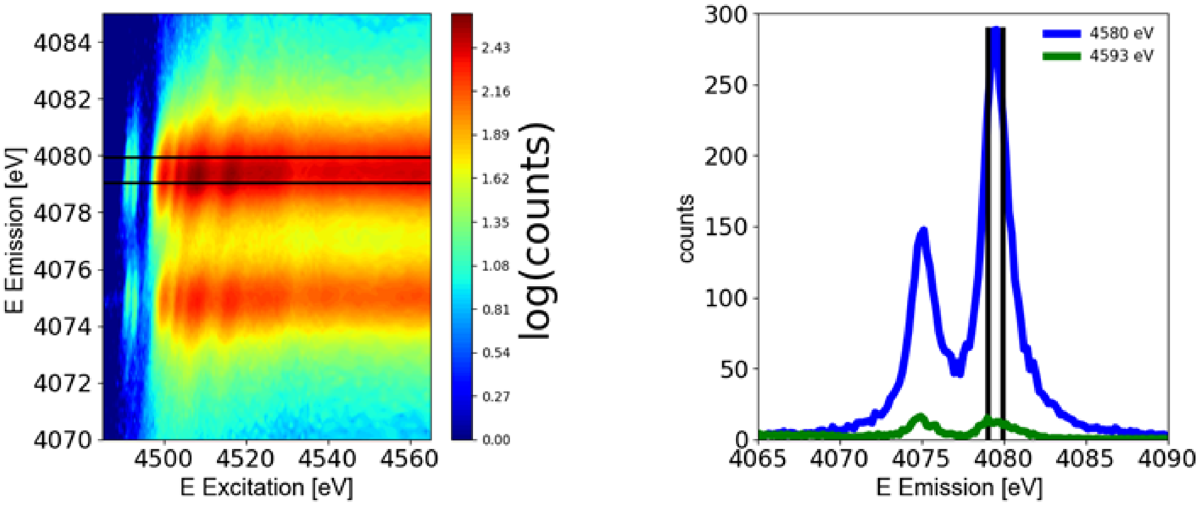

Figure S5: Left: Full RIXS plane at Sc K-edge showing the $K_{\alpha 1}$ (at 4079.5 eV) and $K_{\alpha 2}$ (4074.5 eV) lines. The x-axis shows the energy of the exiting photons while the y-axis the energy of the fluorescent photons. Right: X-ray emission line for the excitation energies 4580 and 4592 eV. The vertical black bars mark the range which was set to derive the HERFD spectra.

The High Energy Resolution Fluorescence Detected x-ray absorption near-edge spectrum (HERFD-XANES) shown in Figure S6 was calculated from the full RIXS plane taken with the von Hamos spectrometer by integrating the center of the Sc $K_{\alpha 1}$ emission line over about 0.9 eV. The correction for overabsorption was applied using the FLUO module as implemented in the ATHENA software.[2] For the HERFD measurements, the von Hamos spectrometer was used. The sample is mounted in the same way as for the XAS measurements and the curved crystal of the spectrometer is mounted in backscattering geometry above the sample. The main axis of the spectrometer is under 90° relative to the incoming beam. Hence, the geometry needs to be defined on a horizontal plane, spanned by the incoming beam and the spectrometer axis. The angle for the incoming beam is, therefore, 90° and the exit angle of the fluorescence photons is defined by the Bragg angle at the Sc K emission line. From this, we arrive with an entry angle of 90° and an exit angle of about 26°. For the total fluorescence yield, the detector and the incoming beam are on a horizontal plane, with incoming photons at an angle of 90° relative to the detector. The sample is mounted under 80° relative to the incoming beam and at 10° relative to the detector.

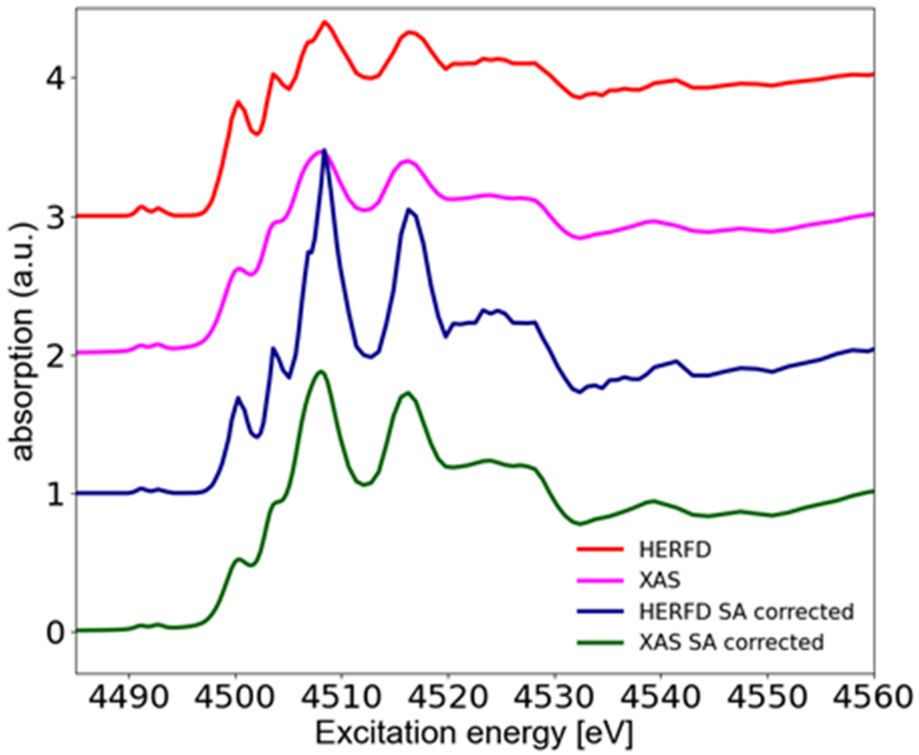

Figure S6: Comparison between HERFD-XANES spectra and a conventional XANES spectrum (XAS) taken from a pellet with pure ScF_3. Dashed lines: raw spectra; solid lines: spectra after correction for overabsorption. Note the more prominent pre-peaks in the HERFD spectra as well as the twofold split of the edge crest at about 4508 eV.

References

(1) Jain, A.; Ong, S. P.; Hautier, G.; Chen, W.; Richards, W. D.; Dacek, S.; Cholia, S.; Gunter, D.; Skinner, D.; Ceder, G.; Persson, K. A. Commentary: The Materials Project: A materials genome approach to accelerating materials innovation. *APL Mater.* **2013**, *1*, 011002, DOI: 10.1063/1.4812323.

(2) Ravel, B.; Newville, M. *ATHENA*, *ARTEMIS*, *HEPHAESTUS*: data analysis for X-ray absorption spectroscopy using *IFEFFIT*. *J. Synchrotron Radiat.* **2005**, *12*, 537–541, DOI: 10.1107/S0909049505012719.
